# A Rare Case of Pediatric Oropharyngeal Schwannoma

**DOI:** 10.7759/cureus.46693

**Published:** 2023-10-08

**Authors:** Mattie Rosi-Schumacher, Maya Raghavan, Michael Pizzuto

**Affiliations:** 1 Otolaryngology, Jacobs School of Medicine and Biomedical Sciences, University at Buffalo, Buffalo, USA; 2 Pediatric Otolaryngology, Jacobs School of Medicine and Biomedical Sciences, University at Buffalo, Buffalo, USA

**Keywords:** schwann cell neoplasm, nerve sheath tumour, head and neck tumor, pediatric otolaryngology, pediatric tumor, laryngeal schwannoma, oropharyngeal mass

## Abstract

This is a case of a male child who presented with a progressively enlarging oropharyngeal mass, hyponasal voice, and symptoms of obstructive sleep apnea. Computed tomography imaging of the head and neck revealed a well-circumscribed low-density area of polypoid morphology arising from the left lateral pharyngeal wall, filling most of the posterior oropharynx and extending to a pedicle at the lateral nasopharynx. Histopathological evaluation following surgical excision revealed Antoni A tissue and S100 immunoreactivity. The presentation and diagnosis of benign schwannoma neoplasms are discussed.

## Introduction

Schwannomas are a type of benign tumor of the nervous system. They grow from Schwann cells in the peripheral nerve sheath and often present as a well-circumscribed mass [1]. Approximately 25% to 45% of schwannomas are found in the head and neck region, most commonly in the parapharyngeal space, face, orbit, oral and nasal cavity, mastoid, and larynx [2,3]. Complete surgical excision is the recommended treatment, although techniques may differ depending on tumor location [4]. Recurrence and malignant transformation are uncommon, but they can occur [5]. Schwannomas typically present in the second to fifth decade of life [6,7]. This is the first published report of a pediatric patient with a schwannoma originating at the lateral wall of the nasopharynx.

## Case presentation

A 14-year-old male with no significant past medical or surgical history presented with a mass in his throat. He had a negative streptococcus test at an urgent care center and was then referred to the emergency department. He complained of two days of sore throat, nasal congestion, and an expanding mass in the back of the mouth. He reported voice changes and odynophagia. He denied difficulty breathing, difficulty opening his mouth, fevers, or referred otalgia. He was tolerating a normal diet. Oral examination showed a swelling in the midline of the posterior oropharynx. A viral panel was positive for respiratory syncytial virus (RSV), but no other laboratory values were obtained. He was diagnosed with bacterial pharyngitis with peritonsillar involvement by the emergency department providers, prescribed antibiotics, and discharged that same day to home. The otolaryngology service was not consulted and did not evaluate the patient during this visit.

The patient presented three months later to the pediatric otolaryngology clinic with complaints of recent voice change and symptoms of obstructive sleep apnea. He denied sore throat, dysphagia, odynophagia, otalgia, dyspnea, neck pain, or trismus. He had been maintaining a normal diet. On a physical exam, the patient was noted to be afebrile, in no acute distress, and breathing comfortably on room air. He had a hyponasal voice. Oral examination revealed moist mucosa, normal healthy dentition, and normal mouth opening. His tongue was midline with normal movement, and there was no asymmetry to the natural arch of the palate. The tonsils were 2+ on the Brodsky scale [8], and the uvula was midline. There was a large, firm, pink, nonerythematous mass obstructing most of the posterior oropharynx. His neck had no masses or palpable cervical lymphadenopathy. The patient was sent from the clinic to the emergency department for labs, imaging, and definitive management.

**Figure 1:**
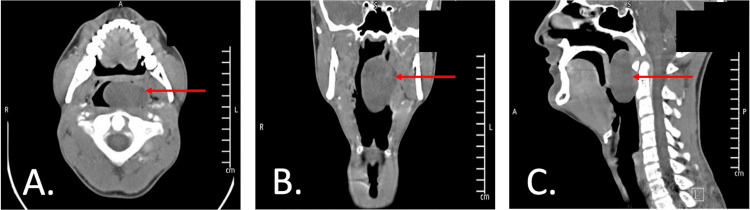
CT images (A. axial B. coronal C. sagittal) Computed tomography imaging of the neck with intravenous contrast shows a well-circumscribed low-density area of polypoid morphology arising from the left lateral pharyngeal wall, filling most of the posterior pharynx with maximum measurements of 2.6 x 3.9 x 5.3 cm.

In the emergency department, labs did not reveal any abnormal values. Computed tomography (CT) of the neck with intravenous (IV) contrast (Figure 1) was obtained, which showed a rounded well-circumscribed low-density area of polypoid morphology arising from the left lateral nasopharynx and occupying most of the posterior oropharynx. There was no thickening or enhancement of the structure’s walls or internal air identified. The maximum measurements were 2.6 x 3.9 x 5.3 cm. The mean internal Hounsfield units measured 39.7. The airway appeared patent, the tonsils were not enlarged, the retropharyngeal space was within normal limits, and the lymph nodes were unremarkable. The patient was admitted to the hospital with plans for surgical excision. 

**Figure 2:**
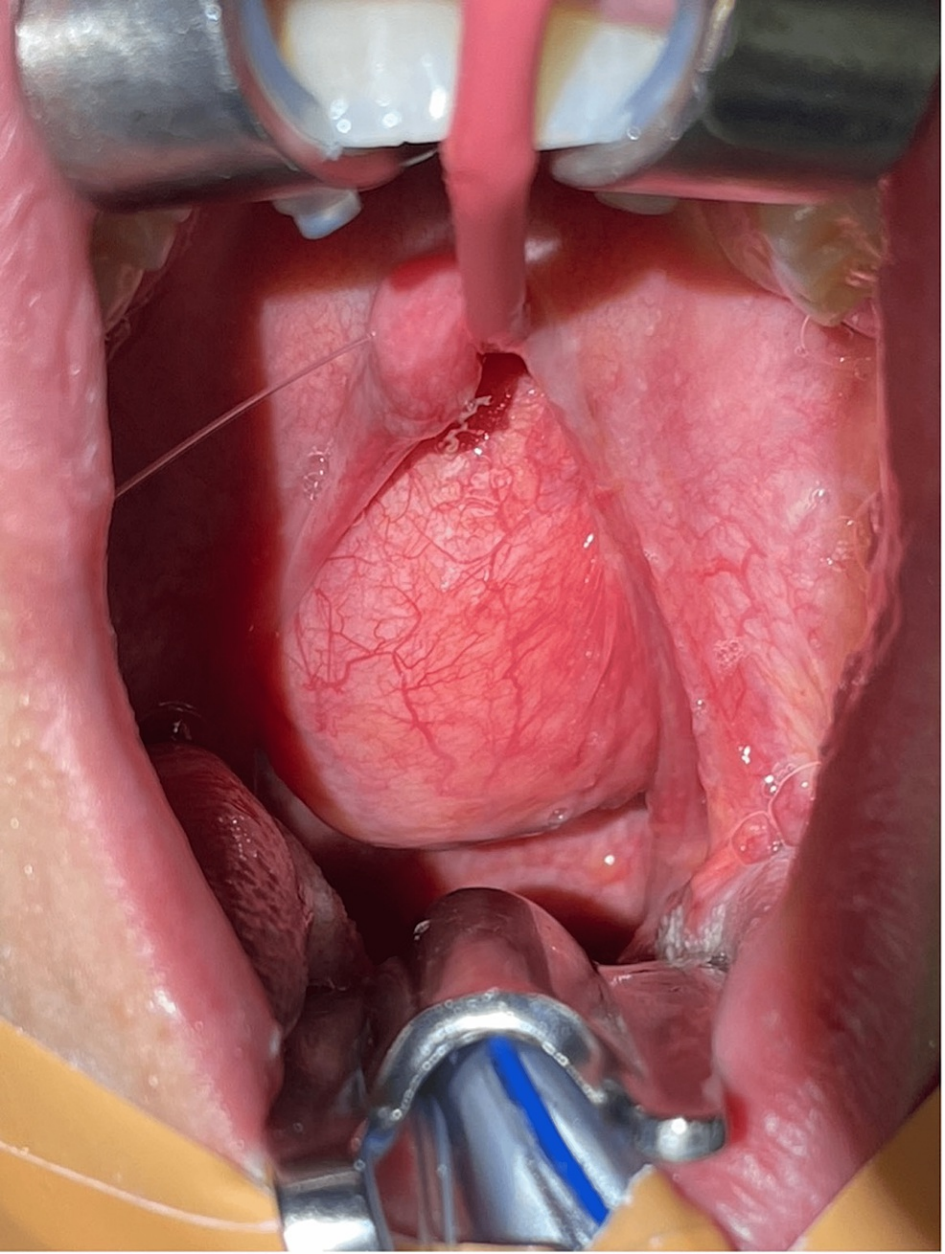
Intraoperative appearance of the mass Intraoperative gross appearance of the oropharyngeal mass occupying most of the posterior oropharynx.

That same day, the patient was taken to the operating room for excision of the oropharyngeal mass. Under general anesthesia, the patient was placed in the Rose position. Exposure was achieved using a Crowe-Davis mouth gag (Medline Industries, Inc., IL) and a red rubber catheter passed through the left nasal cavity and out the oral cavity to retract the soft palate. An obvious tumor was visible obstructing the entire oropharynx extending toward the nasopharynx (Figure 2). The mass was adherent to the left pharyngeal musculature. While inferior traction was applied to the tumor, the mass was separated from the left pharyngeal muscles using blunt dissection and monopolar cautery. The stalk was identified, emanating from the pharyngeal wall just lateral to the left posterior choana. Dissection was then directed from the superior aspect of the attachment to the inferior extent towards the left tonsillar fossa. The mass was excised from the base of the stalk and sent for histologic evaluation. Bleeding at the base of the stalk was controlled with suction cautery. There were no complications. 

**Figure 3:**
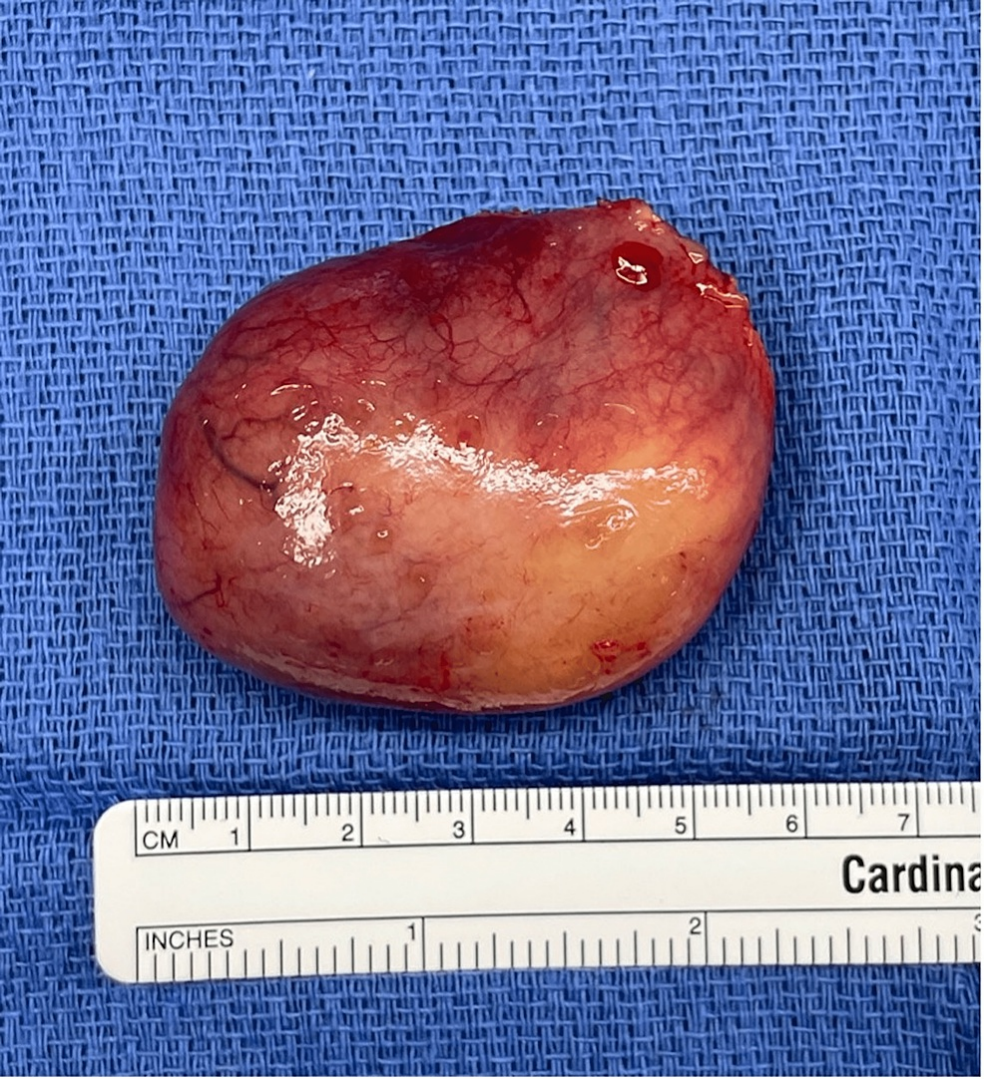
Pathologic specimen Gross appearance of the oropharyngeal mass after surgical excision weighing 40 grams and measuring 5.5 x 4 x 3.3 cm.

The gross specimen pathology report revealed a pharyngeal mass described as an oval tan-pink solid mass encased in a thin translucent fibrous capsule, weighing 40 grams and measuring 5.5 x 4 x 3.3 cm (Figure 3). There was no hemorrhage, necrosis, or cystic changes seen.

**Figure 4:**
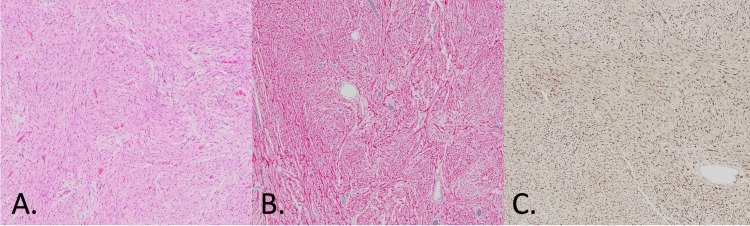
Histopathological slides Histopathological slides of the lesion magnified at 10x demonstrating (A) compact Antoni A-type tissue on H&E staining, (B) S100 immunoreactivity, and (C) SOX-10 immunoreactivity.

Histopathological examination of the lesion showed compact Antoni A-type tissue with scattered foci of degenerative nuclear changes using hematoxylin and eosin (H&E) staining (Figure 4A). The neoplastic tissue exhibited extensive S100 (Figure 4B) and SOX-10 (Figure 4C) immunoreactivity. There was no evidence of malignancy. The mass was diagnosed as a schwannoma.

The following day after surgery the patient was noted to be recovering well and tolerating a regular diet. He was breathing comfortably, and his pain was controlled with Tylenol and ibuprofen. Physical examination showed the posterior oropharynx was clear without evidence of bleeding and minimal erythema over the left anterior tonsillar pillar. He was discharged home in stable condition.

## Discussion

Typically, 25%-45% of schwannomas occur in the head and neck region and are most common between the second and fifth decade of life [2,6]. The majority of schwannomas in this region are reported to occur in the parapharyngeal spaces [2]. Most schwannomas are sporadic, while rare cases can be associated with neurofibromatosis type I or II [9]. An estimated 50% of parapharyngeal schwannomas originate from the vagus nerve, with the next most common site being the cervical sympathetic chain [10]. Pediatric schwannomas are rare and account for less than 10% of all schwannomas [11]. There is only one published case report by Zhu et al. documenting a pharyngeal schwannoma in a pediatric patient, which originated from the posterior oropharyngeal wall and measured 2.5 cm in size [7]. Our report represents the first published case of a pediatric patient with a schwannoma arising from the lateral wall of the nasopharynx and measuring greater than 5 cm in maximum dimension.

Schwannomas of the head and neck region often present as asymptomatic grossly visible masses or with localized obstructive symptoms such as dysphagia, globus sensation, voice changes, or difficulty breathing [12-16]. Patients often delay presentation for care for many months, and initial misdiagnosis commonly leads to further delay in treatment [6,17]. As in this case, nonspecific symptoms were mistaken for a routine infection by the emergency department providers and led to a misdiagnosis and delay in definitive management. An otolaryngology consultation at the time of this patient's initial presentation may have prompted an earlier diagnosis. The otolaryngologist's expertise in the head and neck exam as well as an earlier referral for head and neck imaging would have been helpful in expanding the clinical workup of this patient's complaints.

Many clinicians report that schwannoma is not in their initial differential diagnosis when they evaluate an intraoral or pharyngeal tumor [6,17]. Intraoral schwannomas are most often mistaken for other mesenchymal tumors or epithelial tumors [18]. Several conditions that are important to consider when evaluating a mass in the pharynx include a salivary gland tumor, neurofibroma, paraganglioma, hairy polyp, or other neurogenic or malignant tumor [12]. Diagnosis of head and neck schwannomas usually includes imaging and pathological evaluation to differentiate them from other possible tumors. Computed tomography (CT) and magnetic resonance imaging (MRI) are used initially to inform clinical decision-making and can be expected to show well-circumscribed soft tissue lesions [5].

Surgical excision is the standard treatment for schwannomas, and there is typically a low rate of recurrence or complications [4]. Although the nerve of origin is rarely identified, excision without nerve isolation has been shown to be safe with a low risk of neurologic complications [12,19]. The fibrous capsule and hypovascularity typical of schwannomas aid in safe excision, although surgery can be more complex depending on tumor location [4]. The definitive diagnosis of schwannomas relies on post-excision histopathological analysis [5,20]. The diagnosis is confirmed with microscopic examination demonstrating Antoni A and B areas and Verocay bodies along with expression of S100 on immunohistochemistry stains [21].

## Conclusions

This case presentation is unique due to the young age of the patient and size of the tumor, which led to near-total obstruction of the posterior oropharynx. The diagnosis of schwannoma should be considered in a pediatric patient presenting with an enlarging mass in the oral cavity and complaints of voice changes, dysphagia, or obstructive symptoms. Early CT imaging of the head and neck will aid in a more timely diagnosis, and referral to an otolaryngologist should be made for definitive surgical management.
